# Supplementary material for: Are carbon emissions trading and green financial instruments synergistic? -Comprehensive quantitative research based on content analysis
Source: PLoS One. 2024 Mar 7;19(3):e0298601. doi: 10.1371/journal.pone.0298601 (PMC10919601; doi:10.1371/journal.pone.0298601)
Supplement: S1 Data — (ZIP) [file pone.0298601.s001.zip › Data/地级市Z_F工作报告.zip!/║┌┴·╜¡/╞δ╞δ╣■╢√╩╨/└┤╫╘╣1⁄2╓┌║┼í╛┬φ┐╦╩2╛▌═°í┐_2013.docx]

各位代表：

　　现在，我代表市人民政府，向大会作工作报告，请予审议，并请各位政协委员提出意见。

　　一、2012年工作回顾

　　2012年是新一届政府的开局之年。面对国际金融危机持续影响、国内经济增速放缓的严峻形势，在市委的正确领导下，我们牢牢把握稳中求进的总基调，大力推进产业项目建设，着力优化经济结构，全面加快城市建设步伐，切实保障和改善民生，全市经济社会实现了平稳发展。预计地区生产总值实现1180亿元，同比增长8%；财政总收入完成118.6亿元，下降16.1%；全社会固定资产投资完成677亿元，增长32%。

　　——产业项目建设实现新突破。全市重点推进总投资5000万元以上产业项目184个，其中超亿元项目148个。年度开复工项目156个，完成投资177亿元，是近年来产业项目建设数量最多、力度最大、效果最好的一年。装备制造业项目34项，完成投资45亿元。新建了一拖大型收获机械和水田机械项目，龙马重工、德达机械、柒远兴轮毂等12个超亿元配套项目加快推进，腾翔铸锻造完成设备安装调试。农业和矿产资源开发项目51项，完成投资53亿元。中粮生化能源玉米深加工、佐源菊苣深加工、港进马铃薯食品科技产业园等项目取得新进展，龙江杏山铜矿开发和依安陶瓷产业基地项目加快建设，徽记休闲食品、华润雪花啤酒等项目建成投产。新兴产业项目14项，完成投资17亿元。中恒集团医药产业及物流园项目落地开工，红嘴生物科技、天维大豆浓缩蛋白等项目启动实施，龙源风电场、蓝天能源生物天然气等项目进展顺利，安泰乳酸链球菌素项目竣工投产，清华同方信息港项目正式签约。深入实施园区建设“15211”工程，投入4.2亿元用于基础设施建设，园区吸纳和承载项目能力明显增强，新入驻产业项目24个。争取批次用地指标1162公顷，项目建设用地得到保障。

　　——工业经济稳中有进。坚持“上大扶中抓小”，主动应对危机，工业经济在逆境中实现稳定增长。规模以上工业完成增加值270亿元、主营业务收入860亿元、利税95亿元，分别增长10%、11.5%和8.5%。装备工业优化升级，齐轨交通建成全国首台铁路货车整车疲劳与振动实验台，北满特钢产能由80万吨提升到130万吨，一拖大农机销售大中型拖拉机、高速插秧机、喷灌设备2800台（套），龙华新能源汽车顺利下线。我市高端装备制造业被认定为“国家科技兴贸创新基地”。食品工业优势初显，飞鹤、北大仓、博天、瑞盛等食品工业企业保持增势，规模以上食品工业增加值增长14.5%，占规模以上工业增加值的34.5%。支持中小企业发展，出台了《关于加快中小企业发展的意见》，对30户成长性好的中小企业给予重点支持；为15户企业补办了房屋产权证，新增银行授信额度5.6亿元；出资7000万元规范壮大中小企业融资平台，48户企业获得助保金贷款5亿元。科技创新成果显著，取得科技成果145项，获省级以上科学技术奖20项；高新技术产业实现产值587.5亿元，增长8%。品牌战略深入实施，中国驰名商标和地理标志证明商标达到14件，7种产品获国家地理标志保护产品。节能减排工作全面推进，万元GDP综合能耗下降3.7%，水环境质量明显提高，城市空气质量二级以上天数稳定超过340天。

　　——农村经济形势喜人。农业增加值实现283亿元，增长11%。粮豆薯总产246.5亿斤，增长14.5%，再创历史新高，实现“五连增”目标。种植结构不断优化，玉米、水稻和马铃薯种植面积分别达到1838万亩、386万亩和215万亩；牧草、杂粮和蔬菜种植面积达到210万亩，其中洋葱种植面积8万亩，产量占全国的1/4。畜牧业健康发展，实现产值172亿元，增长10%。奶牛存栏68万头，增长10%，继续保持全省第一；鲜奶总产量162万吨，增长11%。农业科技化、产业化、组织化水平有新提高。兴十四现代农业示范园区二期工程扎实推进，县级农业科技园区发展到24个。新增省级农业产业化龙头企业14户，农产品加工转化率达到38%，提高3个百分点。马铃薯产业初步形成，种薯繁育、贮存、销售、深加工规模不断扩大。新增农民专业合作社550个，总数达到4160个，带动36.3万农户，人均增收1560元。新发展千万元以上现代大型农机合作社34个，总数达到191个，田间综合机械化程度达到90%。土地规模经营1345万亩，增长6.7%，占全市耕地面积的44.4%。新转移农村劳动力3.5万人，新增劳务收入5亿元。农业基础设施得到加强。大力实施“节水增粮行动”，新增高效节水灌溉面积60.8万亩、水田46万亩，增产粮食3亿斤。加固堤防119.4公里，除险加固水库37座。植树造林34.5万亩，治理水土流失45.6万亩。争取土地整理专项资金15亿元，占全省的1/3。秋整地大面积推进。耕地保护工作连续十年居全省第一。农村集体土地所有权确权登记发证工作基本结束。

　　——第三产业繁荣活跃。第三产业实现增加值422亿元，增长6.8%。商贸物流业加快发展。百花小商品商务中心一期工程开工建设，奥特莱斯、居然之家、南海国际大酒店和兴隆大家庭等新型商业业态签约入驻。铁锋综合物流产业园一期项目启动建设，汽贸城已有10家4S店入驻营业。建华恒腾物流园区投入运营。“万村千乡”市场工程稳步推进，汽车、家电、餐饮消费继续攀升。社会消费品零售总额实现480亿元，增长15%；进出口总额完成8亿美元，按可比口径增长31.8%。旅游业持续升温。扎龙温泉小镇、扎龙景区停车场主体工程完工，飞鹤乳业生态观光牧场加快建设，湿地博物馆、北大仓酒文化园和满族风情园等项目完成规划设计。城际旅游联盟合作继续深化。央视《江山多娇》栏目对扎龙美景进行了专题报道。“十一”旅游黄金周接待游客突破150万人次，创历年新高。旅游总收入实现104亿元，增长21%。金融业不断壮大。各项存款余额突破千亿元大关，达到1122亿元，比年初增加140亿元；各项贷款余额753亿元，增长18%，超过全省平均水平。与国家开发银行签订了“十二五”开发性金融合作备忘录。举办大项目银企对接活动，签订意向性贷款协议146亿元，目前到位82亿元。浦发银行落户我市。新增小额贷款公司13家、担保公司2家。大力发展直接融资，启动“区域集优债务融资”，恒阳牛业、东北变压器、瑞盛食品3户企业纳入首批中小企业直接债务融资债券发行范围；民生证券成为我市金融战略合作伙伴；安泰生物、华工机床等企业上市工作加快推进；加大注资力度，优化资本结构，城投公司融投资能力进一步增强，成功发行第二期企业债券15亿元，完成大项目投资32亿元。我市入选中国金融生态城市。会展业有新突破。成功举办绿博会、小交会、奶业博览会及首届扎龙国际婚礼文化节、啤酒文化节、齐齐哈尔鹤文化艺术节等重要会节，实现交易额130亿元，增长10%。

　　——城乡建设全面提速。统筹搞好新城开发、老城改造和村镇建设，城乡建设迈出新步伐。修编了 2010-2020年城市总体规划，并通过国务院部级联席会议审查。完成了城市综合交通、中心城区加气站、全市中小学分布等11项专项规划。“一城三线”建设全面推进，新城启动项目25个，御园、金域名都和紫金华府3个高档小区开工建设，国际五金建材城和商务会所主体工程落成，市中级人民法院审判中心完成基础施工，市公安指挥中心主体封顶，哈齐客专563、565铁路跨线桥基本建成；“通航滨水线”劳动湖进出水船闸完成主体施工，4座景观桥梁建设顺利，新立街桥和安顺路桥已实现临时通车；“迎宾门户线”中华路实施路面改造6.6公里，新建景观节点8处；“品牌旅游线”齐扎公路文化旅游节点项目加快建设。成功引进中汇城、恒大、中冶等大型知名开发企业，商品房建设档次得到提升。保障性住房建设不断加快，全市改造棚户区106.4万平方米、16591户，开工建设回迁房135.4万平方米、17176套，1.3万户迁入新居；建设廉租房300套、公租房400套。棚改工程“畅心园”、“清馨雅居”被评为“中国和谐社区创新示范项目”和“中国保障性安居工程示范楼盘”，安居工程建设受到央视专访。改造城市旧小区6个，完成既有建筑热计量改造35万平方米、低温楼改造56.6万平方米。改造农村泥草房3.94万户、264万平方米，农村住宅砖瓦化率达到73.8%，提高3.3个百分点。投资9.2亿元，新开工及续建“三供两治”项目47项。齐扎、齐梅公路建成通车，公路客运枢纽、北苑货运枢纽、齐昂公路及公铁立交桥启动建设。改造、新建市政道路和打通“卡脖路”、“断头路”13条。村镇建设取得新进展，11个省百镇建设试点镇完成投资9.57亿元，建设项目84个；5个城中村开工建设回迁房15.4万平方米；实施整村推进25个。兴十四镇被授予省级可持续发展实验区。城市管理“四级网络”体系初步建立，相对集中处罚权正式实施，城市管理精细化、规范化、市场化水平有新提高。集中整治中华路、卜奎大街等重点街路，进一步加大城市净化、亮化和绿化力度，拆除违章建筑（构筑物）5000处，实施亮化104处，城区绿化62.5公顷，城市形象和品位明显提升。

　　——社会事业长足进步。统筹推进社会事业，不断完善服务体系，公共服务水平进一步提高。教育事业均衡发展。新建和改扩建公办幼儿园30所，新增义务教育标准化学校48所，改造危房校舍30.8万平方米。中职毕业生就业率达到95%以上。市职教中心、铁路工程学校、卫校成为国家级示范校。发放助学金5019万元，惠及学生4.7万人次。我市荣获全国教育改革创新优秀奖和“两基”工作先进单位。医疗卫生服务能力不断增强。医药卫生体制改革深入推进，群众就医用药负担有所减轻。基层医疗卫生服务水平逐步提高，2家社区卫生服务中心被评为国家级示范中心，村卫生所达标率70%。结核医院门诊楼投入使用，市第一医院南院、中医院综合楼建设顺利。深入实施人口计生“惠家工程”，圆满完成国家流动人口均等化试点任务。公共文化服务体系逐步健全。国有文艺院团改制顺利完成。风情音画《达斡尔人》在全国第四届少数民族文艺会演中获得11项大奖，动画系列片《神鹤丹丹》制作完成，马戏团3支表演团队在国外演出950余场次。文化惠民工程深入实施，新建农家书屋186个、乡镇文化站14个，全市156处公共文化设施全部免费开放。国民体质监测健康指导中心顺利建成。成功举办了亚洲女子冰球锦标赛、全国青年女子篮球联赛等赛事。我市运动员在伦敦残奥会上获得两金一铜。三中女排在亚洲少儿运动会上获得第三名。

　　——群众生活不断改善。30件民生实事基本落实。城镇居民人均可支配收入17404元，农村居民人均纯收入8876元，分别增长14.5%和14.9%。居民储蓄存款余额781亿元，增长14.1%。我市被评为中国十佳优质生活城市。就业形势总体平稳。深入实施创业带就业工程，城镇新增就业11.24万人，下岗失业人员再就业9.54万人，就业困难人员就业4.2万人，城镇登记失业率3.88%。社会保障体系更趋完善。新型农村和城镇居民养老保险试点实现全覆盖，新农保参保率达80%，新增城镇职工基本养老保险2.6万人，离退休人员养老金人均月增210元。新农合参保率99.35%，新入校学生全部纳入医保，城镇职工基本医保和居民医保最高支付限额分别提高了33%和2倍，城镇职工和居民基本医保住院报销比例高于全省11.2和6.4个百分点，29万困难企业退休人员及灵活就业参保人员纳入特殊慢性病医保范围。失业保险基金实现市级统筹，参保48.56万人，待遇标准提高12%。厂办集体企业改革在全省率先启动。建立城市居民低保标准动态调整和收入信息核对机制，城市低保标准月人均提高到301元，农村低保标准年人均提高到1718元。农村五保集中供养率达61%。环卫工人待遇有所提高。社会福利事业稳步推进，新建残疾人康复站56个，SOS儿童村青年公寓、第二社会福利院综合楼投入使用。居民出行更加便利，与中国联合航空公司合作开通至北京航线运行良好，新开通至韩国首尔国际航线；中心城区新增出租车700台，公交车延时线路增至10条。扶贫开发稳步推进， 2.7万农村人口实现脱贫。

　　——社会管理持续加强。扎实推进“平安鹤城”建设，全面提升社会管理科学化水平，全市社会更加和谐稳定。社会治安综合治理成效显著。城市技防信息化建设不断加强，科技主导警务模式基本形成，治安管控水平大幅提升。继续推进“警力下沉”，“阳光警务”深入实施。大力开展侦破命案、“打四黑除四害”、打击经济犯罪和毒品犯罪等专项行动，成功侦破一批大案要案，群众安全感和满意度进一步提高。“六五”普法和法治城市创建活动扎实开展，社区矫正和安置帮教工作取得成效。安全监管力度进一步加大。严格落实安全生产责任制，深入整治重点领域安全隐患，安全生产形势持续稳定好转。开展了以食品、农资为重点的市场监管联合执法，市场秩序更加规范。创新食品安全监管模式，强化食品安全量化分级管理，食品安全工作经验在全省推广。信访工作得到加强。深入开展领导干部接访、带案下访和重访治理专项行动，全力推进群体访积案化解工作，信访案件办结率明显提高。社区建设和管理步入新阶段。进一步完善物业属地化管理机制，物业管理水平有新提高。社区公益用房三年发展规划全面完成，城市社区公益用房平均面积达到680平方米，提前实现省定目标。新建国家和省级综合减灾示范社区9个。社区网格化服务试点扎实推进。社区工作者生活待遇有较大提高。农村社区建设启动率达70%，建成样板社区19个，拜泉县国富镇德润社区荣获全省“十佳和谐社区”。

　　——行政效能明显提高。全国首创的行政执法平台正式开通，首批进入行政执法平台部门26个，投诉举报案件办结率100%。新政务中心投入使用，电子监察系统配套建立。全面启动以“三集中、三到位”为重点的行政审批方式改革，49个部门的448项行政审批事项集中进驻办理，事项集中办理率达77.4%，行政审批效率大幅度提高。进一步明确了市区管理权限，下放事权30项，调整事权7项。对房产管理、城市管理、公安管理、安全生产监管、食品安全监管等体制进行了改革与调整。政府机构改革、机构编制核查和事业单位清理规范工作全面完成。自觉接受市人大和市政协监督，人大代表议案和政协委员提案办结率均达100%。起草审修地方性法规2部，制定出台政府规章3部。廉政风险防控体系初步建立。《齐齐哈尔行政问责暂行办法》制定出台。审计工作得到加强。

　　民族宗教、机关事务、外事侨务接待、新闻出版、社会科学、民兵预备役、妇女儿童、地方志、“双拥”、统计、档案、人防、地震、气象等工作取得了新成绩。

　　各位代表！2012年我市经济社会发展取得的成绩来之不易。这是省委、省政府和市委正确领导的结果，是市人大、市政协和社会各界有力监督、鼎力支持的结果，是全市各级政府和广大干部群众同心同德、团结拼搏的结果。在此，我代表市人民政府，向全市人民，向所有关心、支持齐齐哈尔发展的各界人士，表示崇高的敬意和衷心的感谢！

　　在看到成绩的同时，我们也清醒地认识到，全市经济社会发展和政府工作还存在一些问题。由于国际经济复苏缓慢和国内经济下行压力较大、国内外市场需求不旺、财政基金收入大幅减少等因素影响，我市2012年地区生产总值、财政总收入没有实现预期目标。主要是：产业层次不高，经济结构不优，抗御风险能力不强；县域经济总量偏小，工业化、城镇化进程缓慢；工业园区建设滞后，牵动发展的大项目、好项目不多，对税收贡献率较低；财源结构不合理，增收乏力。社会管理还需加强，改善民生的任务还比较繁重。部分机关干部廉洁自律意识不强，“吃拿卡要”现象还不同程度地存在，经济发展环境、行政效能仍需改善和提高；一些领导干部为民服务和谋发展、抓落实的能力有待进一步增强。对此，我们一定高度重视，认真加以解决。

　　二、2013年工作任务

　　2013年是全面深入贯彻落实党的十八大精神的开局之年，是实施“十二五”规划承前启后的关键一年，是为全面建成小康社会奠定坚实基础的重要一年。全市经济社会发展既面临很多困难和挑战，同时也具备很多有利条件和积极因素。一方面，世界经济复苏缓慢，欧债危机带来的不利影响还在加剧，国际大宗商品市场剧烈震荡，国内经济增速趋缓的可能依然存在。受宏观经济形势影响，我市装备制造、冶金、化工等传统行业生产经营艰难的状况短时间内难以改善。另一方面，党的十八大确定了新的发展目标，全国正在掀起全面建成小康社会的热潮，我国经济必将实现新一轮跨越式发展。国家继续实施积极的财政政策和稳健的货币政策，加大基础设施建设、改善民生等方面的投入，城市化、城乡一体化进程将进一步加快。国家大力发展战略性新兴产业，扶持小微企业发展，为我市产业结构调整、小微企业成长壮大提供了难得机遇。特别是我市一批产业项目相继开工建设，农业连年丰收，商贸流通繁荣活跃，城乡居民收入稳定增长，全市上下干事创业、加快发展的信心和动力明显增强。只要我们紧紧抓住这些宝贵的发展机遇，充分调动各方面积极性，凝心聚力，埋头苦干，我市经济社会就一定能够实现健康快速发展。

　　政府工作的总体要求是，深入贯彻党的十八大和中央、省委经济工作会议精神，全面落实科学发展观，按照市委十二届二次全会总体部署，以城市化、城镇化和城乡一体化为统领，牢牢扭住经济建设不放松，突出抓好产业项目建设，把改善民生、增加城乡居民收入作为一切工作的出发点和落脚点，解放思想，改革创新，稳中求进，努力实现经济社会更好更快发展，为全面建成小康社会奠定坚实基础。

　　主要预期目标是，全市地区生产总值增长11%以上；财政总收入增长15%以上；全社会固定资产投资增长30%以上，力争超过35%；规模以上工业增加值增长12%以上；社会消费品零售总额增长15%以上；城镇居民人均可支配收入增长12%以上；农村居民人均纯收入增长13%以上；城镇登记失业率控制在4.5%以内；万元GDP综合能耗下降3.5%。

　　为实现上述目标，重点做好以下工作：

　　（一）以推动经济发展实现新跨越为目标，全力打好项目建设攻坚战。按照市委“项目攻坚年”部署，坚持以县（市）区为主体，以产业项目为重点，深入实施定向定位招商和小分队招商，确保新引进项目（含续投项目）投资到位额增长20%以上。重点推进固定资产投资5000万元以上产业项目超过230个。围绕壮大县域经济总量上项目。以项目建设推进农业现代化、新型工业化、农村城镇化和城乡一体化，促进县域经济发展实现重大突破。全市新开工固定资产投资5000万元以上工业项目达到2012年的3倍，力争到“十二五”末，基础较差的县实现GDP 100亿元以上，基础较好的县实现GDP 300亿元以上。依托优势资源开发项目。充分利用我市农畜产品富集的优势，统筹谋划、建设一批投资规模大、科技含量高、税收贡献多、带动能力强的大项目、好项目，力争新上投资超亿元农业产业化项目25个以上。搞好石油、高岭土、铜矿、石材、风能、地热、天然苏达水等资源开发利用规划，实现科学、有效、持续开发，用资源换企业、上项目、立产业，变资源优势为经济优势。延伸产业链条生成项目。依托装备制造、食品加工等重点产业链条，结合市场需求，通过大企业裂变、产业链延伸上项目，形成产业集群、企业集聚、配套发展的新格局。建好园区承载项目。坚持县域工业园区化，制定出台支持园区建设的政策措施，积极发展一批特色鲜明、错位发展的专业化园区。以齐齐哈尔国家高新区为引领，集中力量把“15211”工程建设的5个园区建成全市重点产业集中区，新上亿元以上项目不少于20个。富拉尔基工业园区争取晋升国家级经济开发区。加快龙沙、铁锋、建华、昂昂溪4个中小企业园区建设，推进中小企业集聚发展。加快推进依安、富裕、泰来、讷河、克东、碾子山等特色园区建设。完善相关政策，发展“异地经济”，实现优势共享。完善机制推进项目。建立健全更加务实管用的项目建设组织领导机制、目标责任机制、服务保障机制、督办考评机制、奖惩问责机制，完善“走、比、看”活动，将项目建设的数量规模、投资额度、进展程度、经济效益等定标纳入年度目标考核体系。创新项目建设包保、服务和推进方式，对项目洽谈、签约、引进、开工、建设、投产及后续服务等环节实施全程包保，专项专办，特事特办，着力解决项目建设遇到的困难和问题，保证项目无障碍建设。

　　（二）以调整优化结构为主线，实现工业经济速度、效益同步提升。加快信息化和工业化深度融合，以信息化带动工业化，进一步转方式、调结构，推动传统产业与新兴产业、大企业与中小企业共同发展，确保工业经济稳中有进、好中有快。支持大企业做大做强。以信息化为引领，加快一重、北钢、齐重数控、齐二机床、昊华、飞鹤、博天、博城等老企业技改扩能和新产品研发，通过提高传统产业信息化水平扩大市场份额、赢得更大效益。继续加强与央企总部联系，为大企业争取更多支持。加强对重点行业和重点企业的监控，强化煤、电、油、气、运和原材料调度服务，确保大企业稳定生产。装备制造、绿色食品、煤油化工产业主营业务收入分别增长8%、20%和15%。促进中小企业总量扩张。把发展中小企业作为富民、就业的重要渠道，认真落实好国家、省和我市关于促进中小微企业发展的一系列政策措施，本着“非禁即入”的原则，全力推进中小微企业大发展、快发展。实施产业配套工程，引导和支持中小企业与大企业协作配套，加快发展3-5个产值达数百亿元的配套企业群。实施“小巨人扶持培育计划”，市级重点扶持成长性好的中小企业100户以上，每个县（市）区重点扶持10户以上。推动瑞盛食品与上海光明集团合作，鼓励、支持我市优势产品打入高端市场。完善中小企业服务中心功能，实行“一站式”、“窗口式”服务。非公经济增加值占GDP比重达到55%以上。加快新兴产业成长壮大。积极培育发展新兴产业，加大对新投产项目的扶持力度，优化经济结构，增强发展后劲，尽快打造引领全市工业经济发展的支柱企业。支持一拖大农机引入、开发核心技术，提升产业竞争力；支持齐轨交通货车、龙华新能源汽车扩大生产，提高市场占有率；支持中恒药业、清华同方信息港加快建设。战略性新兴产业主营业务收入增长22%。强化科技支撑能力。制定促进科技创新体系建设实施意见，引导企业加快科技创新，建立科技成果超市，推进科技成果落地生成和新技术集成应用。高新技术产业增加值占GDP比重达到13%以上。深入实施品牌战略，巩固国家商标战略实施示范城成果。

　　（三）以发展农民专业合作组织为支撑，全面推进现代化大农业建设。坚持以推进土地规模经营为基础，以发展农民专业合作社为重点，加快提高农民组织化水平，有效聚集整合农业农村各种资源和要素，促进农业集约化、标准化、产业化发展和农民分工分业，为实现农业现代化和农村城镇化夯实基础。加快组织化步伐。按照农民专业合作社“两年内全覆盖”的要求，新建一批，规范一批，提升一批，确保农民专业合作社发展到6800个，其中新组建现代大型农机合作社20个。加强入社农民和理事会成员培训，促进合作社规范化、标准化建设。坚持走“公司+合作社”的路子，积极探索“龙头企业+合作社+农户+银行+担保公司”五位一体的“三农”服务模式。推进土地规模经营，面积达到1614万亩，增长20%。新转移农村劳动力4万人。大力发展畜牧专业合作社，突出抓好以奶牛、肉牛为重点的园区化规模养殖，扩大紫花苜蓿和青贮玉米种植面积，提高畜禽养殖科技含量，加强畜产品质量安全管理，促进畜牧业提档升级。提高产业化和科技化水平。发挥农业龙头企业带动作用，提高农产品附加值。加快苏州悦华大豆精深加工、台湾嘉壹香生猪屠宰加工、天津海皇马铃薯精深加工等农业产业化项目建设，力争农产品加工转化率达到45%以上。支持兴十四现代农业园区尽快建成国家级示范园区，县级以上农业科技园区发展到25个。积极培育引进优良品种，大力推广高产栽培技术模式，加快农业科技化进程。完善农业基础设施。抢抓国家和省加大水利建设投入的有利机遇，加快尼尔基水库下游配套、高效节水灌溉、水库除险加固及中小河流治理等8个重点工程建设，新增高效节水灌溉面积50万亩、水田30万亩。推进龙江花园水库、乡镇供水、嫩江干流堤防等9个项目建设。加大深松整地力度，强化基本农田和土壤地力保护。大力发展高产高效农业。深入实施“2513”工程，玉米、水稻、大豆、马铃薯、甜菜等主要作物高产创建核心区面积超过200万亩、示范辐射区面积超过500万亩、带动区面积超过1000万亩，为“十二五”期末粮食产能达到300亿斤以上创造条件。加快绿色（有机）食品产业发展，新建国家级绿色食品原料基地30万亩。大力发展杂粮产业，种植面积达到150万亩，打造全国杂粮产业基地。抓好“菜篮子”工程，支持棚室蔬菜生产，加快蔬菜基地建设，力争达到65万亩。做大马铃薯产业，延伸产业链条，叫响种薯品牌，种薯面积达到30万亩。积极发展洋葱产业，提高产品质量和市场占有率。

　　（四）以增强区域经济发展活力为着眼点，大力发展第三产业。提升传统服务业，培育现代服务业，重点发展生产性服务业，切实提高服务区域经济发展的能力。打造区域商贸物流中心。统筹发展全市商贸物流业。以大型连锁企业集团为载体，构建便民服务的传统商贸网络；以中汇城为载体，打造高端商贸，积极发展新型物流；以百花商务中心为载体，壮大小商品物流；以富尔农艺为主要载体，依托种子公司和各种子经销站（店），谋划建设种子生产交易物流中心。加快铁锋综合物流产业园、北苑货运枢纽和中恒医药产业及物流园建设，完善各类专业市场，增强辐射能力，全力打造农资、农产品、农机、五金、建材、汽车等多点开花的区域性商贸物流中心。扩大对外贸易，优化出口结构，提高非公经济出口比重，深化对俄边贸合作。进出口总额增长10%。发展壮大旅游业。以文化为魂，强化特色文化与旅游项目的融合，丰富旅游业的文化内涵，促进旅游业全面提升。打造扎龙5A级景区，加快建设扎龙温泉小镇、飞鹤乳业生态观光牧场、北大仓酒文化园和满族风情园等齐扎公路文化旅游节点，建好湿地博物馆，搞好野生动物园、飞碟射击靶场、旅游房车营地等项目规划论证并抓紧实施，充分彰显我市独具特色的历史文化、鹤文化、湿地文化、酒文化和绿色文化。继续搞好昂昂溪罗西亚大街、碾子山自然风光、梅里斯民族风情等特色旅游开发。引进战略投资者，靠市场化运作发展壮大旅游业。办好关东文化旅游节和扎龙国际婚礼文化节。加快旅游名镇建设。促进会展经济繁荣活跃。提升绿博会、小交会办会质量和层次，促进房展、车展、服装展等各类展会蓬勃发展，实现会展业品牌化、专业化和产业化。创新会展方式，推动展会由政府主导向以市场为主、现场交易向订单交易转变。引入外地知名会展，进一步活跃会展经济。加快“走出去”步伐，积极参加国内外各类展会。力争会展业成交额增长10%以上。

　　（五）以城乡规划、建设和管理为抓手，加快推进城市（镇）体系建设。坚持“三化”建设既定目标，高起点规划，高标准建设，高效能管理，突出抓好以“一城三线”为重点的中心城区建设，统筹推进县城、小城镇和新型农村社区建设，构筑现代新型城市（镇）体系，提高城镇化率。突出规划的引领和约束作用。注重统筹协调，启动至2030年市域总体规划修编工作，做优城市总体规划、小城镇规划和新型农村社区规划，完善规划体系。注重科学完备，做细交通、建筑、“三供两治”、城市照明、电网建设等专项规划，增强城市功能。注重风格特色，做好文化、生态、滨水等标志性城市景观规划设计，提升城市品位。强化规划执行，切实维护规划的严肃性和权威性。掀起大开发大建设热潮。坚持新建与改造并举，形成大建设、快改造、严监管的工作局面，推动城乡面貌大改观。一是全力推进新城建设。抓好居然之家、奥特莱斯品牌折扣店、星级高档酒店、生态休闲娱乐购物广场、金融写字楼等中汇城项目建设，推进市公安指挥中心、中级人民法院审判中心、一重总部和研发中心、公路客运枢纽、高铁新站等12个公建项目建设，加快新城建设速度。二是优化住房结构。建立保障性住房、商品房互为补充的住房供应体系，满足群众住房需求。改造棚户区6495户、45.69万平方米；建设廉租房200套、1万平方米；建设公租房200套、1.2万平方米。改造城市旧小区7个、既有建筑热计量30万平方米、低温楼50万平方米、农村泥草房4万户。开发城市精品小区20个。三是开展“市政基础设施建设年”活动。加快城区地下污雨水管网、供热管网、天然气加气站、生活垃圾卫生填埋场、集中热源建设和城市道路升级改造，完成劳动湖景观桥梁、引水明渠等重点工程。科学设计并加快实施中华路、卜奎大街、龙华路立面改造及重要节点建设。抓好齐昂公路及公铁立交桥建设，力争2014年10月建成通车。加快建设南北高速互通区、南外环等交通工程。继续推进农村公路建设，完善路网布局，抓好管理养护。四是加快县城和村镇建设。抓好县城建设，力争在新城区和产业园区建设上有新跨越。抓好省百镇建设试点镇、市级示范镇规划建设和城中村改造，完善配套设施和服务功能，统筹解决好进城农民的户籍、就业、保障等问题，增强产业集聚和吸纳人口能力。建好标准化新型农村社区，促进农村人口向社区集聚。五是加强建筑市场监管。维护建筑市场秩序，保障工程质量安全。强化房地产市场监管，促进房地产市场持续健康发展。提升城市管理效能。深入构建“四级网络”管理体系，推进城市管理重心下移，完善以市级为主导、区级为主体、街道为基础、社区为前沿的大城管格局。落实城市管理三年工作目标责任制，加强城市管理考核与评价，实现城市管理精细化、规范化、人性化、市场化、数字化和社会化，推动城市管理迈上新台阶。开展城市管理专项会战、主要交通节点秩序整顿和城乡环境综合整治，进一步完善城区环境卫生、冬季清雪等管理机制，巩固扩大“三优两提升”文明城创建成果。强化生态文明建设。把生态文明建设放在突出位置，全面促进资源节约，提供更多生态产品，努力建设美丽鹤城。以建设国家级园林城为目标，重点对12条主要街路、9处公园绿地进行绿化升级，城区绿化25.2公顷。完成三北防护林五期工程建设，植树造林21万亩。治理水土流失45万亩。强化湿地保护，维护生态平衡。加强土地资源节约集约利用和矿产资源合理开发。积极推广新能源汽车，开通新能源汽车城市公交示范线路。加快发展农村清洁能源，抓好公共机构节能。下大力气推进主要污染物减排工作，加快城镇污水处理厂减排工程建设，强化机动车尾气治理和畜禽养殖污染防治，坚决完成年度减排指标。切实抓好废旧商品回收利用工作，基本构建起技术先进、高效利用、生态环保的再生资源回收利用体系。

　　（六）以开源节流为重点，突出抓好财税金融工作。坚持发展骨干财源，培育新兴财源，强化税收征管和金融支撑，努力壮大地方财政实力。在财税方面，重点在“生财”上下功夫，同时做好“聚财、用财、理财”文章。围绕产业抓财源，进一步完善“异地经济”财税分成体制，加快建设一批税收贡献率高的大项目、好项目，培育新的财源增长点。建立现代化税收征管体系和新型纳税服务体系，切实加强和改进税收征管，严厉打击偷逃抗骗税行为，确保各项税收应收尽收。充分发挥财政资金的杠杆作用，吸引各类社会资金参与我市开发建设。不断提高公共财政保障能力，保证重点项目、重点领域支出需求，保证民生领域投入逐年提高。加强财政预算管理和政府投资项目监督审查，严格控制一般性财政支出，提高财政资金使用效益。加强国有资产监管和保值增值工作。在金融方面，着力加大金融对经济发展的服务和支撑力度，促进金融与地方经济共赢发展。丰富金融产品，拓展服务领域，加大对重点项目、中小企业、农民专业合作社的信贷投放力度，扩大金融支持经济发展的辐射面。落实好与国家开发银行签订的开发性金融合作备忘录，在园区建设、项目建设、企业扩张、现代服务业发展等方面争取更多支持。加强银企对接，鼓励企业以股权融资、项目融资、债券融资、设备租赁融资等方式筹集资金。推动哈尔滨银行尽快入驻营业，积极引进中信、兴业、广发、招商、韩亚等外埠金融机构，构建金融大格局。加快发展村镇银行、小额贷款公司、融资性担保公司和新型农村经济合作组织。加强信用体系建设，营造诚信金融环境，防范化解金融风险。推进“区域集优债务融资”和中小企业助保金贷款工作，拓宽企业融资渠道。加快安泰生物、恒阳牛业、华工机床、富尔农艺、瑞盛食品等企业上市进度。做大做强城市融投资平台，提升融资能力。大力激活民间资本，积极引进外来资本，实现融资主体多元化。

　　（七）以促进公共服务均等化为核心，加快发展社会事业。完善服务体系,增强供给能力，保障人人享有基本公共服务。努力办好人民满意教育。大力实施素质教育，坚持立德树人，着力培养学生的实践能力、创造能力和就业能力。继续实施学前教育三年行动计划，进一步满足适龄儿童入园需求。大力推进义务教育学校标准化建设和教育教学信息化建设，扩大普通高中优质资源，不断提升整体教育水平。加强国家级中职示范校建设，筹建职业教育综合实训基地和青少年综合实践基地。加强面向地方发展需要的专业学科建设，推动高等教育提升办学质量。推进合作办学和集中办学，促进城乡教育一体化发展。搞好校长、教师培训，建设高素质、专业化教师队伍。加快发展医疗卫生事业。深化医药卫生体制改革，落实国家基本药物制度，切实保障群众基本就医用药需求和安全。推进区域医疗卫生服务中心建设，建立全科医生培训基地，加快第一医院、中医院、卫校和医学院附属二院、三院等重点工程建设，全面提升医疗救护服务水平，增强辐射能力。继续完善疾病预防控制体系，提高社区卫生服务中心服务能力，加强突发公共卫生事件应急体系建设。大力开展爱国卫生运动，增进群众身心健康。抓好优生优育，提高人口素质，兑现独生子女父母奖励政策。推动文化大发展大繁荣。全面实施文化建设振兴工程，深化文化体制改革，加快制定文化产业扶持政策，促进文化事业和文化产业共同发展。深入实施农村文化“五项重点”工程，完善公共文化服务体系。推进公共文化场馆建设和免费开放，丰富群众业余文化生活。加强文艺精品创作，完善风情音画《达斡尔人》剧目，着力打造一批具有地方特色、反映时代风貌、省内外知名度高的文化精品。繁荣文化演艺市场，搞好马戏团、小剧场话剧常态化演出。办好鹤文化艺术节，打造主题文化。推进鹤文化和动漫、剪纸、满绣、蛋雕基地等重点文化产业项目建设，突显特色文化。强化文化遗产和重点文物保护，扎实推进国家级历史文化名城创建工作。认真落实全民健身条例，提高全民身体素质，塑造体育名城、健康之城品牌。建设射击综合训练馆，改造鹤城体育场，筹办冰上艺术表演活动，积极申办国内国际体育赛事。

　　（八）以改善民生为根本，切实加强社会保障和管理。抓好民生工程，创新社会管理，切实办好改善民生的实事，努力让群众过上更好生活。突出抓好就业。坚持以发展促就业、以创业带就业，加快完善城乡统一的人力资源市场和公共就业服务体系，为城乡劳动者就业创业提供均等化服务。加强建设领域农民工工资支付监管，确保不拖欠农民工工资。加大小额担保贷款发放力度，为失业人员自主创业发放贷款2亿元。积极开展就业援助，充分发挥百花园大学生创业基地等平台示范引领作用，带动更多人创业就业。城镇新增就业9万人，就业困难人员就业3万人。加快构建普惠型社会保障体系。扩大城乡养老保险覆盖面，增强养老保险基金支撑能力，确保养老金按时足额发放。加快医疗保险市级统筹，逐步实现缴费标准、信息系统、待遇和监管统一。继续抓好工伤、失业、生育保险参保工作。扎实推进厂办集体企业改革，积极争取补贴资金和政策支持，切实保障职工合法权益。完善城乡低保标准动态调整、社会救助标准与物价上涨联动和农村五保供养标准增长机制，提高综合救助水平。大力发展社会福利和慈善事业，强化残疾人社会保障和服务体系建设，抓好市儿童福利院、富拉尔基托老服务中心、昂昂溪精神病人疗养院综合楼、第一社会福利院失能老人护理楼项目建设。完善养老扶持优惠政策，鼓励民办养老机构发展，推进民办养老机构连锁、加盟经营，进一步做好为老服务工作。抓好100个扶贫开发整村推进，实现整村脱贫。加大对革命老区贫困村扶持力度。切实提高社区管理水平。加强物业管理，进一步强化县区、街道、社区物业管理主体责任和业主委员会服务功能，建立健全物业企业准入、整顿和退出机制，提升物业管理服务质量。推行社区网格化管理，促进社区规范化建设。建立社区公益用房持续增长机制，城市社区公益用房总面积增加10%。推进农村社区建设，启动率达到80%，创建样板社区10个。稳妥推进乡（镇）区划调整。全力维护社会和谐稳定。加强社会治安综合治理，推进城市技防监控和群防力量向居民小区延伸，严厉打击各类违法犯罪活动。深化“六五”普法，加快法治城市创建步伐。全面推行社区矫正工作。继续保持安全生产监管强势，严格落实安全生产属地管理责任和企业主体责任。以整治校车安全为重点，深入开展“打非治违”专项行动，切实加强特种设备安全监管，有效防范各类安全事故。做好国家级“安全社区”试点工作。完善市场监管联合执法机制，加强食品药品安全监管，严厉查处违法违规行为，进一步规范市场秩序。全面提升12315“一线通”维权功能，切实维护消费者权益。健全“大调解”体系，加快信访三级视频网络平台建设，加大信访责任追究和依法治访力度，力争把历史遗留的重大群体访疑难案件全部化解。

　　强化统计监测。加强民族宗教、机关事务和档案管理。做好外事侨务、妇女儿童、民兵预备役、新闻出版、社会科学、地方志、“双拥”、人防、气象等各项工作。

　　三、切实加强政府自身建设

　　新的一年，全市经济社会发展任务繁重而艰巨。做好政府工作，必须进一步加强政府自身建设，着力打造一支政治强、业务精，懂管理、会招商，作风硬、敢担当的公务员队伍。

　　（一）为民执政，提升政府凝聚力。把群众满意不满意作为检验工作的唯一标准，牢固树立公仆意识、服务意识和责任意识，对群众充满感情，对工作勤勉尽责，对事业毫不懈怠。认真贯彻中央“八项规定”，改进工作作风，密切联系群众，以扎扎实实的工作赢得民心。认真搞好调查研究，坚持轻车简从，严禁迎来送往，真正深入到群众中间，问政于民，问需于民，问计于民，使政府提出的发展思路、作出的工作部署、出台的政策措施更加符合实际，更加符合群众愿望。切实精简会议活动和文件简报，着力在改进会风和文风上作出表率。

　　（二）科学理政，提升政府创造力。以转变政府职能为核心，深入推进行政体制改革，建设职能科学、结构优化、廉洁高效、人民满意的服务型政府。强化新政务中心运行管理，加强县乡政务中心建设，进一步抓好流程再造、审批服务、电子监察等关键环节，努力打造流程最优、服务最佳、时限最短、监管最严的政务服务体系。推进公共资源交易改革，真正做到“一场式交易、一站式服务、一体化监管”。继续加强市政府门户网站行政执法平台建设，力争将所有行政执法部门全部纳入，真正做到让权力在阳光下运行。强化市对区事权下放与调整的督促检查，适时调整完善事权运行机制，确保各项事权真正下放到位、调整到位。

　　（三）高效施政，提升政府执行力。完善重大事项公众参与、专家论证和集体决定相结合的决策机制，促进行政决策科学化、民主化和制度化。对正确的决策，咬定青山不放松，持之以恒抓落实，确保政府工作定一件干一件、干一件成一件、成一件满意一件。严格执行《齐齐哈尔行政问责暂行办法》，坚决克服本位主义、部门利益等不良倾向，大力整治行政不作为乱作为、工作不落实、配合不协调现象，重点解决“中梗阻、底梗阻、勒拿卡”等突出问题，确保政令畅通，真正把广大公务人员的心思凝聚到干事业、办实事上，精力集中到抓落实、促发展上。

　　（四）依法行政，提升政府公信力。严格按照法律、制度、规则和程序办事，做到依法行政、规范执法、公正执法、文明执法，全力构建法治政府。自觉接受市人大及其常委会法律监督和工作监督，大力支持市政协履行政治协商、民主监督和参政议政职能，广泛听取社会各界意见和建议。讲信用，守承诺，着力打造诚信政府。从市政府班子做起，带头遵守《廉政准则》，严格执行廉洁自律各项规定。加强廉政风险防控工作，从源头上预防腐败。强化行政监察和审计监督，严厉查处各种违纪违法行为，坚决纠正各种不正之风，树立为民、务实、清廉的政府形象。

　　各位代表！加快鹤城振兴，全面建成小康社会，是全市人民的热切期盼，是时代赋予的神圣使命。让我们乘着党的十八大的东风，在市委的正确领导下，同心同德，顽强拼搏，共同创造齐齐哈尔更加幸福美好的未来！
